# Acknowledgement to Reviewers of *Biosensors* in 2019

**DOI:** 10.3390/bios10010005

**Published:** 2020-01-16

**Authors:** Biosensors Editorial Office

**Affiliations:** MDPI, St. Alban-Anlage 66, 4052 Basel, Switzerland

The editorial team greatly appreciates the reviewers who have dedicated their considerable time and expertise to the journal’s rigorous editorial process over the past 12 months, regardless of whether the papers are finally published or not. In 2019, a total of 140 papers were published in the journal, with a median time to first decision of 16 days and a median time from submission to publication of 46 days. The editors would like to express their sincere gratitude to the following reviewers for their generous contribution in 2019:
Abdallah, AliLou, ShuaiAgrawal, AnantLu, YudongAlfinito, EleonoraLukyanov, PavelAli, AzaharLyutakov, OleksiyAli, MonsurMa, HanbinAL-Naji, AliManagò, StefanoÁlvarez, LorenaMandal, SoumyajitAntoce, Arina OanaMarasso, Simone LuigiArmijo, Juan FranciscoMarozas, VaidotasAshley, JonMarques, M. MatildeAtobe, MasakazuMartini, ElisabettaAzoug, AurélieMartins, Verónica C.Badea Doni, MihaelaMas, JordiBaffa, OswaldoMassad-Ivanir, NaamaBagal-Kestwal, Dipali R.Massera, EttoreBailón, RaquelMehta, SunaliBanchelli, MartinaMelvin, AdamBarberis, AntonioMemisoglu, GorkemBarbosa, Ana IsabelMendez, FranciscoBarfidokht, AbbasMendez, Martin OswaldoBarrea, LuigiMin, RuiBartsch, RonnyMinamikawa, TakeoBelBruno, JosephMinas, GraçaBeni, ValerioMishra, RupeshBertoncello, PaoloMishra, Rupesh K.Bhalla, NikhilMitsui, ToshiyukiBhattarai, Jay K.Miura, YoshikoBlair, Ewen O.Miyazato, HironariBohbot, JonathanMorasso, CarloBonyár, AttilaMoscone, DanilaBorile, GiuliaMoulick, AmitavaBose, DebojitMu, XuanBottari, FabioMunteanu, Florentina-DanielaBykov, AlexanderMyllylä, TeemuByrne, HughNagles, EdgarCalabretta, Maria MaddalenaNajdahmadi, AvidCalamanti, ChiaraNallala, JayakrupakarCampos, RuiNamiesnik, JacekCampuzano, SusanaNawarathna, DharmakeerthiCaneira, Catarina R. F.Nedoma, JanCappello, TizianaNesmelov, Yuri E.Caro, CarlosNeumann, JoachimCarrillo, JenifferNi, MelodyCasalini, StefanoNightingale, AdrianCasteleiro-Roca, Jose LuisNikoleli, Georgia-ParaskeviCastro Do Amaral, GustavoNilghaz, AzadehCavallari, Marco RobertoNoort, Danny VanCellini, AntonioNovara, ChiaraChang, Chia-ChenNúñez Carmona, EstefaníaChaturvedi, PracheeNyman, JeffryChen, Liang-BiOćwieja, MagdalenaCheng, Ji-YenOrglmeister, ReinholdCheng, ZhiqiangOrtis, AlessandroChiadò, AlessandroO'Shaughnessy, DouglasChiappini, AndreaOsma, JohannChiriacò, Maria SerenaPadalkar, SonalCho, Eun JeongPalanisamy, SelvakumarColominas, SergiPalego, CristianoCostantini, FrancescaPalestra, GiuseppeCosta-Rama, EstefaníaPardy, TamásCostello, Ben De LacyPark, Byung-WookCredi, CaterinaPark, EnochCriado Fernández, AlejandroPark, MinDaems, DevinPasternak, GrzegorzDai, JingPauliukaite, RasaDang, VinhPedrero, MariaDaunys, GintautasPereira, EulaliaDavis, FrankPérez-Lamela, ConcepciónDe Boer, Nanne KPeter, Van Der WalDe Marcos Ruiz, SusanaPetrou, PanagiotaDe Souza Gil, EricPiro, BenoitDenoual, MatthieuPolito, LauraDi Nardo, FabioPolo, FedericoDiaz, Sebastian AndresPoltorak, LukaszDominguez, Rocio B.Postma, MartenDos Santos, José C.S.Pruneanu, Stela-MariaDosche, CarstenRadtke, AleksandraDS, Francisco JavierRamsköld, DanielDu, E(Sarah)Rangnekar, AbhijitDundon, WIlliamRao, AshwinEbrahimi, AidaRasheed, TahirEbralidze, Iraklii I.Ray, ParthaEsposito, FlavioReich, JohannesEvtugyn, GennadyRen, QinlongFaisal, Shaikh NayeemRenken, ChristianFateixa, Sara Isabel AugustoRezaei, BabakFavero, GabrieleRichardson, JosephFengchun, TianRobertson, Joseph W.F.Fenton, BrockRodriguez, Natalia M.Fernandes, ElisabeteRoux, Jean-MaximeFernández, AntonioRoy, DhruvajyotiFerraro, DavideRusdiana, DadiFiedler, GoeranSalceda-Delgado, GuillermoFitter, JörgSamiei, EhsanFoguel, Marcos ViniciusSanchez, SophieFudickar, SebastianSarantopoulou, EvangeliaFuehrmann, TobiasScaramuzza, MatteoFunari, RiccardoSchazmann, BenjaminGamella Carballo, MariaSchrittwieser, StefanGangaraju, RajashekharScida, KarenGarcía, TaniaSeichepine, FlorentGartia, Manas RanjanSerafín, VerónicaGaudin, ValérieŠestak, AnamarijaGebicki, JacekSgobbi, Lívia FlorioGheorghiu, EugenShahshahani, AmirhosseinGiungato, PasqualeShimizu, FlavioGoel, PratibhaShrestha, NamitaGorodkiewicz, EwaSilva, Saimon MoraesGorski, WaldemarSimm, RogerGoswami, NirmalSinyukov, AlexanderGoto, TakaharuSistla, SrinivasGriffith, HenrySomoza, Mark M.Grossi, MarcoSon, JongsangGüntner, AndreasSong, WuzhouGutiérrez, Juan ManuelSophocleous, MariosHalder, AvikSquella, Juan ArturoHan, Soo DeokSriram, RashmiHärmä, HarriSteglich, PatrickHassan, Sammer-ulStrömberg, TomasHavran, LudekSuddala, Krishna ChaitanyaHe, JiayuanSun, MengHenriques, JorgeSzékács, InnaHeo, JinseokSzweda, RozaHeveran, ChelseaTaborri, JuriHorejs-Hoeck, JuttaTarasov, AndreiHuang, Chih-ChiaTavares, AnthonyHung, Chung-ChihTeixeira, Sofia RodriguesHuotari, MattiTemiz, YukselHwang, NathanielTeodorescu, FlorinaIgnatova, TetyanaTernon, CélineIonita, MarianaTian, BoIqbal, Hafiz M. N.Tischler, DirkJakieła, SławomirTomasi, TomJakubowska, MałgorzataTonacci, AlessandroJauset, MiriamTorres-Ramírez, EduardoJeerapan, ItthiponTravagliati, MarcoJemere, AbebawTurkyilmaz, SerolJenkins, KennethUberti, DanielaJi, ShaofeiUeda, HiroshiJoensson, HaakanUjihara, MasakiJolly, PawanUntersmayr, EvaJoshi, TanmayaVan Dam, AnnemiekeJuan-Colás, JoséVanegas, Diana C.Kakabakos, SotiriosVarriale, AntonioKalogianni, DespinaVazquez, Rebeca MartinezKang, TaejoonVidic, JasminaKang, WenjingVillarreal-Gómez, Luis JesúsKant, KrishnaVrabec, TinaKassal, PetarVrentas, Catherine E.Kaur, HarleenVullings, RikKerman, KaganWagner, PatrickKhan, FaheemWalia, SumeetKhrustalev, VladislavWang, JianfengKim, Jong-hoonWang, QingqingKitahama, YasutakaWang, SaiKokkinos, ChristosWang, ShangKomarova, Natalia V.Wang, YanyanKonijnenburg, MarioWeller, Michael G.Kraft, ThomasWennmalm, StefanKrause, Hans-JoachimWojciechowski, AdamKrueger, EddyWozniak-Knopp, GordanaKujawska, MałgorzataXu, JieKulkarni, AtulXu, TingtingKumar, Dinesh KYadav, MarshleenKumar, NareshYang, Chih-ChinKumar, SamirYang, ShengKumar, ShailabhYangui, AymenKusters, IljaYao, Da-JengLang, MichaelYoon, SorahLee, GyudoYu, Feng-YihLee, JaewookYu, JingxianLi, PengZacconi, FlaviaLi, XiangkaiZakaria, A. B. M.Li, ZhengZgarian, Roxana GabrielaLiew, Oi WahZhang, JunLimoges, BenoîtZhang, LeiLin, AbrahamZhang, Ye-WangLin, HongZhang, YiLin, Zuan-TaoZiolkowski, RobertLitti, LucioZolotoukhina, TatianaLiu, WeiluZwergel, ClemensLong, Yuchen


